# Removal Efficiency and Risk Assessment of Polycyclic Aromatic Hydrocarbons in a Typical Municipal Wastewater Treatment Facility in Guangzhou, China

**DOI:** 10.3390/ijerph14080861

**Published:** 2017-08-01

**Authors:** Zhineng Liu, Qing Li, Qihang Wu, Dave T. F. Kuo, Shejun Chen, Xiaodong Hu, Mingjun Deng, Haozhi Zhang, Min Luo

**Affiliations:** 1 Key Laboratory of Water Quality Safety and Protection in Pearl River Delta (Ministry of Education), Guangzhou University, Guangzhou 510006, China; lzn_2015@126.com (Z.L.); Lq15802036371@163.com (Q.L.); hxd60@126.com (X.H.); mingjun0426@163.com (M.D.); zhanghaozhi2008@163.com (H.Z.); gzdxlm1979@163.com (M.L.); 2 School of Civil Engineering, Guangzhou University, Guangzhou 510006, China; 3 Collaborative Innovation Center of Water Quality Safety and Protection in Pearl River Delta, Guangzhou University, Guangzhou 510006, China; 4 Department of Architecture and Civil Engineering, City University of Hong Kong, Hong Kong, China; dave.kuo@cityu.edu.hk; 5 City University of Hong Kong, Shenzhen Research Institute, Shenzhen 518057, China; 6 State Key Laboratory of Organic Geochemistry, Guangzhou Institute of Geochemistry, Chinese Academy of Sciences, Guangzhou 510640, China; chenshejun@gig.ac.cn; 7 School of Environmental Science and Engineering, Guangzhou University, Guangzhou 510006, China

**Keywords:** polycyclic aromatic hydrocarbons, municipal effluents, risk assessment, wastewater treatment, sludge, inverted A^2^/O process

## Abstract

The loading and removal efficiency of 16 US EPA polycyclic aromatic hydrocarbons (PAHs) were examined in an inverted A^2^/O wastewater treatment plant (WWTP) located in an urban area in China. The total PAH concentrations were 554.3 to 723.2 ng/L in the influent and 189.6 to 262.7 ng/L in the effluent. The removal efficiencies of ∑PAHs in the dissolved phase ranged from 63 to 69%, with the highest observed in naphthalene (80% removal). Concentration and distribution of PAHs revealed that the higher molecular weight PAHs became more concentrated with treatment in both the dissolved phase and the dewatered sludge. The sharpest reduction was observed during the pretreatment and the biological phase. Noncarcinogenic risk, carcinogenic risk, and total health risk of PAHs found in the effluent and sewage sludge were also assessed. The effluent BaP toxic equivalent quantities (*TEQ_BaP_*) were above, or far above, standards in countries. The potential toxicities of PAHs in sewage effluent were approximately 10 to 15 times higher than the acceptable risk level in China. The health risk associated with the sewage sludge also exceeded international recommended levels and was mainly contributed from seven carcinogenic PAHs. Given that WWTP effluent is a major PAH contributor to surface water bodies in China and better reduction efficiencies are achievable, the present study highlights the possibility of utilizing WWTPs for restoring water quality in riverine and coastal regions heavily impacted by PAHs contamination.

## 1. Introduction

Polycyclic Aromatic Hydrocarbons (PAHs) are a wide spread class of environmental pollutants of concern [1] due to their carcinogenicity, mutability and toxicity [2]. They originate primarily from incomplete combustion and petroleum spill [3] and are prone to bioaccumulation and biomagnification [4] as well as long-distance transport [5]. Due to their toxicity, PAHs have been listed as contaminants that required monitoring. 16 PAHs have been enlisted for priority monitoring by US EPA and 7 PAHs selected as target PAHs by China National Environmental Monitoring Center [6]. Further, PAH concentration limits have been set by many countries, such as EU [7], US EPA [8], Netherlands [9] and China [10].

Following their release from fuel spillage or atmospheric deposition, PAHs can enter the sewage system through domestic wastewater, industrial discharge, or surface runoff via the urban drainage. In WWTP, PAHs may exist in the dissolved phase or in the sorbed form, attached to sludge or organic-rich suspended solids. The partitioning behavior of PAHs is well understood [11], it is expected that the low molecular weight (LMW) PAHs are mainly in dissolved form whereas the high molecular weight (HMW) PAHs are predominantly bound to organic-rich surfaces or solids.

In China, the growing attention on the pollution of water bodies has led to increasingly stringent sewage discharge standards [12]. WWTPs with conventional treatment process were hard pressed to meet this task with efficiency and economy [13]. The Anaeroic-Anoxic-Aerobic (A^2^/O) treatment process has been widely adopted to address the extensive problem of eutrophication in both freshwater bodies and coastal areas. A^2^/O generally exhibits better performance in nitrogen and phosphorus removal than other treatment processes, probably benefiting from the lower extracellular polymeric substances production in A^2^/O sludge [14]. Given the growing number of A^2^/O treatment facilities in China [13,15], it is important to investigate the fate of PAHs through the A^2^/O process for improving their removal in both existing and new WWTPs.

The aims of the present study were to (1) investigate the loading and removal of dissolved PAHs in a WWTP with inverted A^2^/O process; (2) characterize the profiles of PAHs at different stages of the WWTP; and (3) assess the health risks of PAHs associated with the effluent and the sludge.

## 2. Materials and Methods

### 2.1. Chemicals

A PAH standard solution mix was purchased from o2si Smart Solutions (Charleston, SC, USA). The mix included naphthalene (NAP), acenaphthylene (ANE), acenaphthene (ANA), fluorene (FLU), phenanthrene (PHE), anthracene (ANT), fluoranthene (FLT), pyrene (PYR), benzo(a)anthracene (BaA), chrysene (CHR), benzo(b)fluoranthene (BbF), benzo(k)fluoranthene (BkF), benzo(a)pyrene (BaP), indeno(1,2,3-cd)pyrene (IcdP), dibenzo(a,h)anthracene (DBA) and benzo(g,h,i)perylene (BghiP).

### 2.2. Wastewater Treatment Plant

The studied WWTP was located in Guangzhou, China. It collected combined sewage in canal from the districts of Liwan and Yuexiu and part of the Baiyun district. The core biological treatment was the anoxic-anaerobic-aerobic (Inverted A^2^/O) process which helped to achieve better nitrogen and phosphorus removal. The WWTP had a design treatment capacity of 2.2 × 10^5^ m^3^/d. All tanks and units in the WWTP, except the secondary clarifier, were closed from the atmosphere and have been installed with deodorization devices and ventilation systems.

### 2.3. Sampling

**Figure 1:**
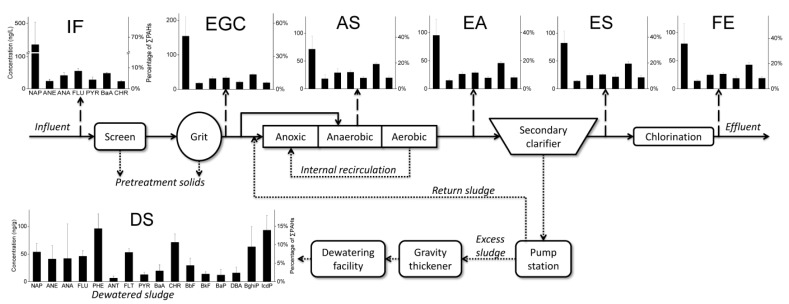
Treatment stages in the WWTP and the sampling points for PAHs. Error bars represent 1 standard deviation of the mean. IN = influent; EG = the effluent of grit chamber; AS = the water-sludge mixture in aerobic stage; EA = the effluent of inverted A^2^/O tank; ES = the effluent of the secondary clarifier; FE = the final effluent after chlorination; DS = dewatered sludge.

Wastewater samples were collected from 6 sampling points (Figure 1) including the influent (IN), the effluent of grit chamber (EG), the water-sludge mixture in aerobic stage (AS), the effluent of inverted A^2^/O tank (EA), the effluent of the secondary clarifier (ES) and the final effluent after chlorination (FE). At each sampling point, 8 grab samples were collected evenly every 3 h to produce an 8-L 24-h composite sample. All collected aqueous samples were stored in amber glass bottle and were filtered immediately. Dewatered sludge samples (DS) were collected from sludge dewatering house and stored at −20 °C in lab. Sampling campaigns were conducted on 4 rain-free weekdays between late November and December 2015.

### 2.4. Chemical Analyses

The wastewater samples were filtrated with 0.7 μm glass fiber filters (Whatman, GF/F) by disc filters. Before use, glass fiber filters were baked at 450 °C in muffle furnace for 5 h to remove trace organic carbon. The filtrated samples (500 mL) were processed by solid-phase extraction (SPE) using an automated extractor (AQUA Trace ASPE799, GL Sciences, Inc., Tokyo, Japan). The SPE stationary phases were conditioned with sequential elution of dichloromethane (5 mL), ethyl acetate (5 mL), methanol (10 mL) and Milli-Q water (10 mL). The samples were passed through the cartridges at a flow rate of 10 mL/min. The cartridges were dried under high purity nitrogen. The analytes were subsequently eluted by ethyl acetate (3 mL) and dichloromethane (3 mL). The combined extracts were evaporated by nitrogen to nearly dry and diluted to 1 mL with ethyl acetate in brown glass vials at 4 °C until chemical analyses. Chemicals used above were all of chromatography grade. The dewatered sludge samples were freeze-dried, ground into powder and passed through 0.2 mm sieve. Sludge PAHs were extracted following the procedure reported in Wu et al. [16].

Sample PAHs concentrations were analyzed in selective ion monitoring mode using Agilent 7890A/5975C (GC-MS, Agilent Technologies, Inc., Palo Alto, CA, USA) equipped with Agilent DB-5MS column (30 m × 0.25 mm (i.d.) with 0.25 μm film thickness, Agilent, Santa Clara, CA, USA). The column temperature program was as follows: temperature increased to 60 °C (1-min hold), 110 °C at 15 °C/min (1-min hold), then successively to 180 °C at 20 °C/min, 203 °C at a rate of 2 °C/min, and 250 °C at a rate of 5 °C/min, and finally to reach 310 °C at 2 °C/min. Pure helium was used as the carrier gas at a flow rate of 1.3 mL/min. 1 μL sample was injected into the injector with an initial temperature of 300 °C using splitless injection mode. The mass spectra were obtained using electron ionization mode at 70 eV and 250 °C.

### 2.5. Quality Assurance and Quality Control

Procedural blanks, spiked blanks (standards spiked into solvent), sample replicates (*n* = 3) and matrix spikes were used to monitor the extraction and analytical quality for the samples. Recoveries of all the PAHs except NAP fell within the range of 65.9 to 111.3%. Recoveries of NAP fell within the range of 38.1 to 51.3%. The relative standard deviations in all replicates were less than 15% as recommended by the US EPA Method 8270D [17]. Instrument detection limits for all individual PAHs were defined as three times of the signal-to-noise ratio.

### 2.6. Toxicity Assessment

#### 2.6.1. Toxic Equivalent Quantity

The combined toxicity of different PAHs in wastewater and sludge samples was evaluated using the BaP toxic equivalent quantity (*TEQ_BaP_*) [18]. This widely applied method [19,20,21] calculates *TEQ_BaP_* using the aqueous phase concentration for the *i*th PAH, *C_i_*, and the prescribed toxic equivalent factors (TEFs) (Table 2; [18]) from the following equation [22]:

(1) $$TEQ_{BaP}=\sum (C_{i}\times TEF_{i})$$

#### 2.6.2. Health Risk

Health risk of PAHs in aqueous samples were assessed following the US EPA health risk assessment guidelines [23] supposing the effluent as source of drinking water for downstream communities. Sixteen PAHs were divided into noncarcinogenic and carcinogenic PAHs for evaluation. Relevant parameters and PAH’s classification are listed in guidance for public health risk assessment [24] and Krishnan et al. [25]. Health risk associated with the carcinogenic PAHs (i.e., BaA, CHR, BbF, BkF, BaP, DBA, and IcdP) was evaluated using Equations (2)–(4) [26,27]:

(2) $$R_{i}<0.01,\;R_{i}=D_{i}\times Q_{i}/70$$

(3) $$R_{i}\geq 0.01,\;R_{i}=\left[1-\exp\left(-D_{i}Q_{i}\right)\right]/70$$

where *R_i_* is annual carcinogenic risk for an individual PAH; *D_i_* is daily intake for carcinogenic PAH (mg/(kg·d)); *Q_i_* is carcinogenic intensity factor; 70 is human average lifetime (a); *C_i_* is concentration of PAH.

(4) $$D_{i}=2.2C_{i}/70$$

where 2.2 is estimated water ingestion rate for adult (L/d); 70 is average weight of adult (kg).

Health risk contributed from the noncarcinogenic PAHs (i.e., NAP, ANE, ANA, FLU, PHE, ANT, FLT, PYR, and BghiP) was assessed as [26,27]:

(5) $$R_{i}=(D_{i}\times 10^{-6}/RfD_{i})/70$$

where 70 is average lifetime; *RfD_i_* is drinking reference dose of noncarcinogen (mg/(kg·d)).

The total health risk, *R*, was determined as the sum of individual PAHs risks [26,27]:

(6) $$R=\sum_{i=1}^{k}R_{i}$$

## 3. Results

### 3.1. PAHs in Dissolved Phase

#### 3.1.1. Comparison between Different Dates

**Figure 2:**
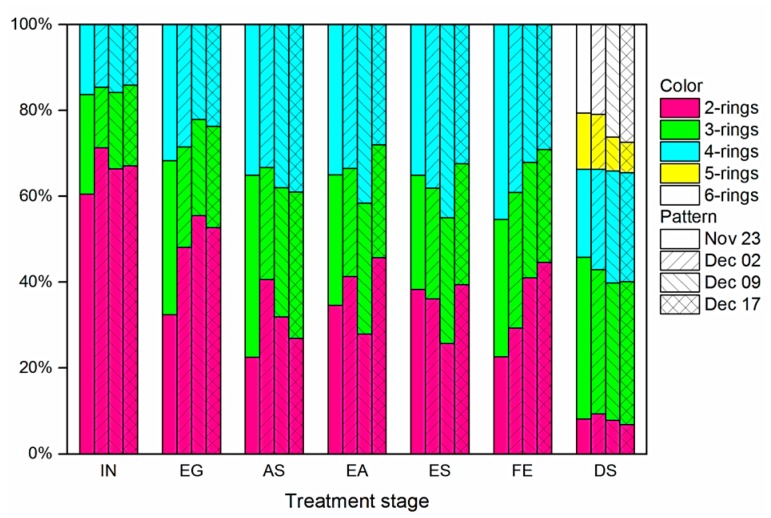
Distribution profiles of PAHs at the different treatment stages.

PAHs concentrations (Table 1) in raw sewage are generally consistent throughout the study, as the level differences across the four sampling dates are small. The influent total PAHs concentration increased by approximately 20% during the first three sampling dates but stabilized by the fourth date. The slight change in influent total PAHs level coincided with transition from autumn to winter in Guangzhou. Similar change in PAHs level has also been reported Qiao et al. [28] and was probably associated with the increase in coal/gas burning for winter heating [29]. The relative distributions of PAHs at various treatment stages also showed little difference over sampling dates (Figure 2).

#### 3.1.2. Concentrations of PAHs

The concentrations of dissolved PAHs at various sampling points are summarized in Table 1 and visualized in Figure 1. Only seven of the targeted PAHs were detected in the aqueous samples; the remaining PAHs were not detected, most likely due to volatilization for the LMW PAHs (i.e., ≤3 rings) and strong sorption for the HMW species (i.e., 4–6 rings). The most abundant PAHs in the influent (IN) was naphthalene (335.66–480.58 ng/L), which accounted for more than 60% of ∑PAHs (554.36–723.24 ng/L). The high levels of IN naphthalene may be attributed to its use as precursor the production of phthalic anhydride, a key starting material for various industrial production [30]. In the effluent samples (FE), ∑PAHs ranged from 189.64 to 262.73 ng/L with naphthalene, again, as the highest component accounting for 22 to 44%. The PAH levels observed here are comparable with those reported for another WWTP at the north of Guangzhou (∑PAHs = 1150 ng/L) [31] but significantly higher than that measured at a Hiroshima WWTP (∑PAHs = 60 ng/L) [32].

#### 3.1.3. Removal Efficiencies of PAHs

**Figure 3:**
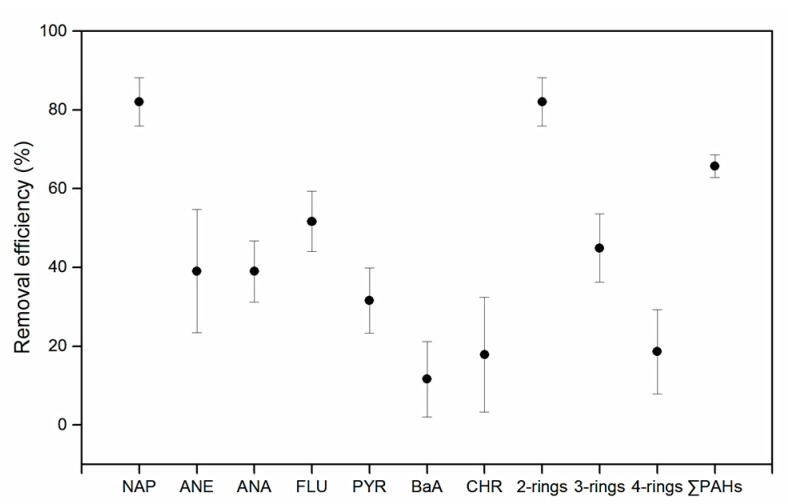
The removal efficiencies of PAHs in the dissolved phase. Error bars represent 1 standard deviation of the mean.

The overall removal efficiencies of ∑PAHs in the dissolved phase through the WWTP was 63 to 69%. The most substantial reduction was observed on naphthalene (2-rings; 60–87%), followed by 3-rings (32–52%) and 4-rings PAHs (3–26%) (Figure 3). The observed overall removal in dissolved phase is comparable to those reported in earlier studies. Ozaki et al. [32] observed a 60% PAHs removal in a Hiroshima WWTP; Bergqvist et al. [33] reported a 30 to 60% removal in a Lithuanian sewer facility. Removal efficiencies as high as 90 to 100% have been observed in specific treatment facilities [33,34] though such performance levels is not common.

**Figure 4:**
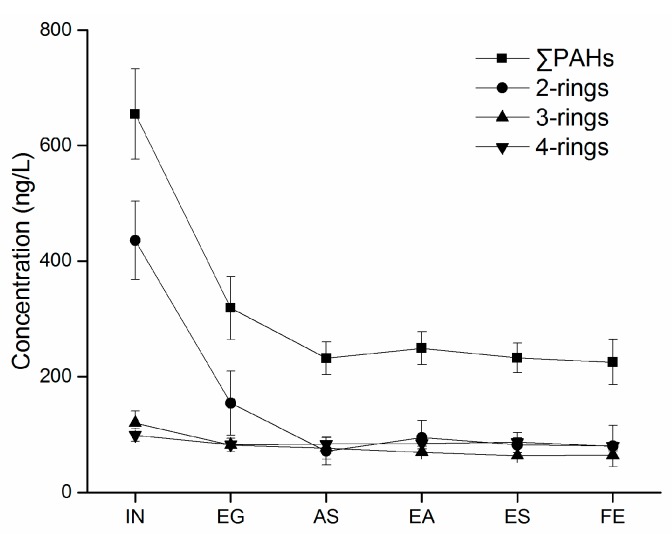
The variation of PAHs at different stages. Error bars represent 1 standard deviation of the mean.

PAH concentrations generally declined along the treatment process (Figure 4). The most significant reduction in PAHs was found during the primary treatment phase (IN-EG) which included fine screen and a grit chamber-clarifier. 50 to 61% of the 2 to 3 ringed PAHs and approximately 46 to 54% of ∑PAHs were removed in this phase. The hydrophobic nature of PAHs, as indicated by their high *K*_ow_, implied that they were more likely to (ad)sorb onto solids rather than remained dissolved [35]. It is generally believed that sorption to particulate and dissolved organic matter, rather than biodegradation, is the major removal mechanism for PAHs during the primary treatment phase [36,37].

During the biological treatment stage (EG-EA), pretreated wastewater was mixed fully with returned sludge and then experiences the anoxic-anaerobic-aerobic (Inverted A^2^/O) process. Reduction in PAHs was mostly attributed to the biological activities of microorganisms. The removal mechanisms include adsorption-precipitation, biodegradation and volatilization [36]. PAHs removal by volatilization was lower than 2% in conventional activated sludge process [36], while the biodegradation effect may also be minor in the absence of microbes selected for PAHs degradation [38].

### 3.2. PAHs in Dewatered Sludge Samples

A summary of the concentrations of PAHs in the dewatered sludge samples are shown in Table 2. All sixteen PAHs were detected in the dewatered sludge samples. Overall, the 3-ringed PAHs (184.40–351.09 ng/L) constituted the most abundant group. This was followed by the 4-ring and 6-ring groups, which together accounted for more than 32% of ∑PAHs (548.06–931.33 ng/L). Phenanthrene was the most abundant 3-ring PAH with concentration ranging from 76.88 to 135.52 ng/g.

The total concentration of the seven carcinogenic PAHs (i.e., ∑PAHs-carc, listed in Table 2) was 200.88 to 308.21 ng/g, accounting for 33.1 to 43.2% of ∑PAHs in dewatered sludge samples. Carcinogenic PAHs are more difficult to biodegrade due to their strong sorption to solids and hence low bioavailability. The higher ∑PAH-carc was found in Guangdong (10 mg/kg) accounting for 33% of ∑PAHs (30 mg/kg), and other cities (average ∑PAH-carc was 7 mg/kg) accounting for 43% of average ∑PAHs (16 mg/kg), according to Cai et al. [39]. Furthermore, the concentrations of 3-rings and 4-rings were also the main contributors to ∑PAH according to Cai et al. [39] and our present study.

### 3.3. PAHs Source Analysis by Diagnostic Ratios

Diagnostic ratios are frequently used to identify the origins of PAHs in environmental [40,41] samples including atmospheric, aqueous, and solid phase samples [16,19,32,42]. The PAHs diagnostic ratios for raw sewage and dewatered sludge are summarized in Table 3. The interpretation of the ratios for the sewage and the sludge samples is generally ambivalent, suggesting the diversity of sources contributing to the PAHs in treatment system. For raw sewage, the light PAHs dominated (i.e., ΣLMW/ΣHMW > 1), suggesting that the sewage PAHs may be petroleum originated [40]. The 5-ring PAH BaA/(BaA + CHR) ratio (0.67–0.69), however, points towards combustion sources. Similarly for the dewatered sludge: ΣLMW/ΣHMW (0.66–0.85), FLT/(FLT + PYR) (0.76–0.88), and IcdP/(IcdP + BghiP) (0.38–0.73) suggest biomass burning as the origin, while ANT/(ANT + PHE) (0.03–0.08) and BaA/(BaA + CHR) (0.07–0.30) hint petroleum sources. The diagnostic ratios may be less appropriate for sewage sludge [43] as PAHs undergo redistribution through partitioning and volatilization, or are selectively enriched/eliminated through the treatment processes. Although the same may not apply to the raw sewage, upstream mixing of different sources can blur the molecular signatures of PAHs [44,45]. Overall, the apparent divergence in these ratios suggests the PAHs collected in the sewage system are likely to have different origins that include petroleum spillage/release and fossil fuel/biomass combustion.

Viewed from another perspective, each diagnostic ratio can be seen as identification only for its containing compounds. For example, the ΣLMW/ΣHMW > 1 suggests that the sewage ΣPAHs may be petroleum originated. The 5-ring PAH BaA/(BaA + CHR) ratio (0.67–0.69) hints that the BaA and CHR may be combustion originated, but not suggests the ΣPAHs sources. Clearly, a PAH diagnostic ratio is valid for corresponding PAHs source identification but not for whole PAHs source identification.

### 3.4. Risk Assessment

#### 3.4.1. Effluent

As WWTP effluent can contribute up to 50% of the total PAHs input to the receiving water bodies, it is therefore necessary to assess the risk associated with the final effluent which would enter into a local river. Since BaP was not detectable in the effluent, the dissolved PAHs were converted to *TEQ_BaP_* following equation (1) for assessment. The effluent *TEQ_BaP_* ranged from 4.23 to 5.27 ng/L, which is approximately 25 times higher than the EU annual average environmental quality standard (AA-EQS = 0.17 ng/L) [7] and the tapwater screening level (3.4 ng/L) [8] proposed by US EPA, but lower than the published maximum allowable concentrations (270 ng/L in Directive 2013/39/EU and 300 ng/L in RCLs).

The personal annual health risk index of detected PAHs in the effluent was shown in Table 4. The total carcinogenic risk ranged from 1.3 × 10^−5^ to 1.6 × 10^−5^/a with benzo(a)anthracene accounting for 99% of the total risk. This is approximately 10 to 15 times higher than the acceptable risk level of 0.1 × 10^−5^/a set by Minister of Health of the People’s Republic of China [46]. Although the concentrations of naphthalene were higher than benzo(a)anthracene, the risk contribution of naphthalene was considerable less as its TEQ is 100 times less than that of benzo(a)anthracene (Table 2).

#### 3.4.2. Dewatered Sludge

Since only BaP and total PAHs are restricted in China, the potential health risk associated with sludge PAHs was assessed using the Netherlands soil standard [9] and the screening level recommended by US EPA [8].

PAHs in the sludge samples generally met the agricultural standard (CJ/T 309-2009) recommended by the Ministry of Housing and Urban-Rural Development of the People’s Republic of China [10]. However, the sludge PAHs levels exceed the Netherlands soil standards [9] and the screening levels recommended by US EPA [8]. The sludge PAHs concentrations generally exceeded the Netherlands standards, with the indeno(1,2,3-cd)pyrene level exceeding by six-fold. When compared with the US EPA screening levels, only benzo(a)pyrene and dibenzo(a,h)anthracene levels exceeded.

For comprehensive evaluation, the *TEQ_BaP_* of 16 PAHs (*TEQ_BaP_*_16_) was calculated. The *TEQ_BaP_*_16_ ranged from 31.38 to 74.38 ng/g in sludge samples, which were 1 to 2 times of the *TEQ_BaP_* of soil standard in the Netherlands (*TEQ_BaP_*_10_ = 32.98 ng/g). Seven carcinogenic PAHs *TEQ_BaP_* (*TEQ_BaP-carc_*) ranged from 30.20 to 72.60 ng/g accounting for 97.5 to 98.1% of *TEQ_BaP_*_16_, which indicated that seven carcinogenic PAHs were main source of risk in sewage sludge.

## 4. Discussion

This work highlights the potential of employing WWTPs as the starting point for remediating/restoring water quality of surface waters in China. Studies have demonstrated that elevated PAH removal efficiencies—as high as 90 to 100%—are possible in conventional sewage treatment facilities [33,34,47]. Recent works have suggested that sewage treatment facilities may be a major contributor of PAHs to surface waters in China. Qiao et al. reported that WWTP effluent accounted for 60 to 90% of the total PAHs input to the receiving rivers within the Haihe River System [48]. Similarly, Qi et al. [49] estimated that 80% of the riverine PAHs in the greater Beijing area may be traced to sewer facilities while Zheng et al. [50] showed that the riverine PAHs levels closely follow those of the upstream WWTP effluents in the Three Gorges Reservoir region in China. This implies WWTPs can be critical in a successful remediation and restoration of water bodies on a local or regional scale. Further investigations on the relative importance of treatment configurations, operating conditions of individual processes/units, as well as environmental factors and influent quality are needed.

## 5. Conclusions

The present study investigated the concentrations and removal of PAHs in a representative WWTP in Guangzhou, China. Seven PAHs (554.36–723.24 ng/L in influent; 189.64–262.73 ng/L in effluent) existed in all dissolved phase samples. Naphthalene dominated over other PAHs in the influent. All 16 US EPA PAHs (548.64–931.33 ng/g) were detected in the dewatered sludge samples, with the 3-ring PAHs as the most dominant group. The removal efficiencies of PAHs from the dissolved phase generally decreases with the number of aromatic rings. The highest removal efficiency was observed for naphthalene. Reduction in PAHs was most effective in the primary treatment phase followed by the core biological process.

Risk assessments suggested that the dissolved phase PAHs concentrations were within the maximum acceptable level while those of the dewatered sludge samples exceeded the environmental standards adopted by The Netherlands and US. Carcinogenic PAHs constitute the main source of risk in both the effluent and the sludge samples. Overall, the assessment exercise underlines the urgent need to lower carcinogenic risks associated with treated sewer and sludge.

## Figures and Tables

**Table 1:** Concentration of PAHs at various stages of sewage treatment (ng/L).

Date	Stage	2-Rings	3-Rings			4-Rings			∑LMW PAHs	∑HMW PAHs	∑PAHs
		NAP	ANE	ANA	FLU	PYR	BaA	CHR			
23 November	IN	335.66	21.06	50.82	56.49	19.14	48.06	23.13	464.03	90.33	554.36
	EG	85.15	19.78	38.33	36.13	16.14	46.17	20.87	179.39	83.18	262.57
	AS	55.91	29.16	39.83	35.95	15.36	47.90	23.70	160.85	86.96	247.81
	EA	86.19	18.41	30.51	26.41	14.95	49.47	22.44	161.52	86.86	248.38
	ES	100.19	15.25	29.27	25.03	15.54	51.04	25.04	169.74	91.63	261.37
	FE	43.51	11.13	28.07	22.37	14.73	49.18	23.44	105.08	87.35	192.44
2 December	IN	449.82	16.64	28.88	43.49	22.43	47.45	22.05	538.84	91.93	630.77
	EG	142.31	16.16	24.46	28.46	21.53	41.39	21.27	211.39	84.19	295.58
	AS	103.53	14.86	24.19	27.51	22.37	44.11	18.42	170.09	84.90	254.99
	EA	108.20	14.04	22.62	29.25	19.62	46.94	21.04	174.11	87.60	261.70
	ES	84.94	13.97	20.88	25.70	29.07	42.15	18.21	145.50	89.44	234.93
	FE	55.66	13.69	20.83	25.34	16.24	39.18	18.70	115.52	74.12	189.64
9 December	IN	480.58	27.31	41.74	59.41	36.59	51.87	25.75	609.04	114.21	723.24
	EG	217.03	18.90	31.12	37.77	24.83	43.08	18.33	304.81	86.24	391.05
	AS	73.94	14.53	26.56	28.68	21.56	45.19	21.04	143.70	87.79	231.49
	EA	58.82	13.36	22.95	27.69	21.83	45.34	20.14	122.81	87.32	210.13
	ES	51.19	12.87	21.71	23.77	21.04	46.37	22.07	109.55	89.49	199.04
	FE	107.75	17.02	25.19	28.54	24.24	42.54	17.46	178.49	84.23	262.73
17 December	IN	475.85	29.38	43.63	60.99	33.21	45.57	20.71	609.85	99.48	709.33
	EG	171.78	16.11	29.97	30.73	20.77	40.36	15.81	248.59	76.94	325.53
	AS	51.55	13.77	24.80	26.77	18.63	40.18	15.95	116.90	74.76	191.66
	EA	126.31	13.49	28.28	30.83	20.97	40.54	15.76	198.91	77.27	276.18
	ES	92.23	13.40	25.07	27.36	20.14	40.02	15.64	158.07	75.81	233.88
	FE	113.67	13.58	24.51	28.86	19.21	39.42	15.43	180.62	74.06	254.67

IN = influent; EG = the effluent of grit chamber; AS = the water-sludge mixture in aerobic stage; EA = the effluent of inverted A^2^/O tank; ES = the effluent of the secondary clarifier; FE = the final effluent after chlorination; DS = dewatered sludge.

**Table 2:** Concentration of PAHs in dewatered sludge (ng/g), soil standards (ng/g), and toxic equivalent factors.

Ring	Compounds	Dewatered Sludge				Soil Standards						TEF ^a^	
		23 November	2 December	9 December	17 December	MHSPE ^b^		RCLs ^c^		CJ/T ^d^			
2	NAP	76.10	51.22	45.87	41.53	15		-		-		0.001	
3	ANE	10.48	32.85	58.21	62.44	-		-		-		0.001	
	ANA	134.88	18.80	0.34	13.52	-		-		-		0.001	
	FLU	58.81	48.74	40.28	36.22	-		-		-		0.001	
	PHE	135.52	76.88	84.54	87.32	50		-		-		0.001	
	ANT	11.28	7.13	4.41	2.62	50		-		-		0.01	
4	FLT	59.58	57.47	49.07	45.61	15		-		-		0.001	
	PYR	18.55	7.88	13.21	11.28	-		-		-		0.001	
	BaA ^e^	18.59	4.53	25.49	29.28	20		160		-		0.1	
	CHR ^e^	93.61	58.39	64.74	68.13	20		16,000		-		0.01	
5	BbF ^e^	47.77	29.64	21.72	17.91	-		160		-		0.1	
	BkF ^e^	19.24	9.87	11.60	14.11	25		1600		-		0.1	
	BaP ^e^	26.06	13.37	3.56	4.88	25		16		2000		1	
	DBA ^e^	29.72	17.40	9.53	6.32	25		16		-		1	
6	BghiP	117.91	46.79	43.54	44.13	-		-		-		0.01	
	IcdP ^e^	73.23	67.69	109.94	121.87	20		160		-		0.1	
∑PAHs-carc		308.21	200.88	246.58	262.52	-	-		-		-		
∑LMW PAHs		427.08	235.62	233.65	243.66	-	-		-		-		
∑HMW PAHs		504.25	313.01	352.41	363.54	-	-		-		-		
∑PAHs		931.33	548.64	586.06	607.20	-	-		5000		-		
∑PAHs-carc/∑PAHs		33.1%	36.6%	42.1%	43.2%	-	-		-		-		

^a^ Toxic equivalent factors [18]; ^b^ Soil standard in the Netherlands [9]; ^c^ Resident Soil in Regional Screening Levels provided by USEPA [7]; ^d^ Disposal of sludge from municipal wastewater treatment plant-Control standards for agricultural use (CJ/T 309-2009) [10]; ^e^ Carcinogenic PAHs. LMW = low molecular weight (i.e., ≤3 rings); HMW = high molecular weight (i.e., 4–6 rings); ∑PAHs-carc = the total concentration of seven carcinogenic PAHs.

**Table 3:** Diagnostic ratios of selected PAHs at different samples.

Diagnostic Ratios	Main Source ^a^	Dissolved Phase of Raw Sewage				Dewatered Sludge			
		23 November	2 December	9 December	17 December	23 November	2 December	9 December	17 December
ΣLMW/ΣHMW	<1 (Combustion)	5.14	5.86	5.33	6.13	0.85	0.75	0.66	0.67
	>1 (Petroleum)								
ANT/(ANT + PHE)	<0.1 (Petroleum)	-	-	-	-	0.08	0.08	0.05	0.03
	>0.1 (Combustion)								
FLT/(FLT + PYR)	<0.4 (Petroleum)	-	-	-	-	0.76	0.88	0.79	0.80
	0.4–0.5 (Petroleum combustion)								
	>0.5 (Biomass combustion)								
BaA/(BaA + CHR)	<0.2 (Petroleum)	0.68	0.68	0.67	0.69	0.17	0.07	0.28	0.30
	>0.35 (Combustion)								
IcdP/(IcdP + BghiP)	<0.2 (Petroleum)	-	-	-	-	0.38	0.59	0.72	0.73
	0.2–0.5 (Liquid fossil fuel combustion)								
	>0.5 (Biomass combustion)								

^a^ Adapted from Yunker et al. [40]; - One or two compounds were not detected.

**Table 4:** The carcinogenic and noncarcinogenic risk index of detected PAHs in effluent.

	*Q*	*RfD*	Risk Index			
			23 November	2 December	9 December	17 December
BaA	0.73	-	1.6 × 10^−5^	1.3 × 10^−5^	1.4 × 10^−5^	1.3 × 10^−5^
CHR	0.0073	-	7.7 × 10^−8^	6.1 × 10^−8^	5.7 × 10^−8^	5.1 × 10^−8^
NAP	-	0.02	9.8 × 10^−10^	1.2 × 10^−9^	2.4 × 10^−9^	2.6 × 10^−9^
ANE	-	0.06	8.3 × 10^−11^	1.0 × 10^−10^	1.3 × 10^−10^	1.0 × 10^−10^
ANA	-	0.06	2.1 × 10^−10^	1.6 × 10^−10^	1.9 × 10^−10^	1.8 × 10^−10^
FLU	-	0.04	2.5 × 10^−10^	2.8 × 10^−10^	3.2 × 10^−10^	3.2 × 10^−10^
PYR	-	0.03	7.4 × 10^−10^	5.9 × 10^−10^	6.4 × 10^−10^	5.9 × 10^−10^

*Q* = carcinogenic intensity factor; *RfD* = drinking reference dose of noncarcinogen.

## References

[1] Balcioglu E.B., Aksu A., Balkis N., Ozturk B. (2014). T-PAH contamination in mediterranean mussels (mytilus galloprovincialis, lamarck, 1819) at various stations of the turkish straits system. Mar. Pollut. Bull..

[2] Wu Y., Luo Y., Zou D., Ni J., Liu W., Teng Y., Li Z. (2008). Bioremediation of polycyclic aromatic hydrocarbons contaminated soil with *Monilinia* sp.: Degradation and microbial community analysis. Biodegradation.

[3] Zhang W., Wei C., Chai X., He J., Cai Y., Ren M., Yan B., Peng P., Fu J. (2012). The behaviors and fate of polycyclic aromatic hydrocarbons (PAHs) in a coking wastewater treatment plant. Chemosphere.

[4] Tao Y., Yu J., Xue B., Yao S., Wang S. (2017). Precipitation and temperature drive seasonal variation in bioaccumulation of polycyclic aromatic hydrocarbons in the planktonic food webs of a subtropical shallow eutrophic lake in China. Sci. Total Environ..

[5] Mori J., Sæbø A., Hanslin H.M., Teani A., Ferrini F., Fini A., Burchi G. (2015). Deposition of traffic-related air pollutants on leaves of six evergreen shrub species during a mediterranean summer season. Urban For. Urban Green..

[6] Zhou W., Fu D., Sun Z. (1991). Determination of black list of China’s priority pollutants in water. Res. Environ. Sci..

[7] Commission E. (2013). Directive 2013/39/eu of the european parliament and of the council amending directives 2000/60/ec and 2008/105/ec as regards priority substances in the field of water policy. Off. J. Eur. Union.

[8] US Environmental Protection Agency Regional Screening Levels (Formerly Hhmssl—Human Health Medium-Specific Screening Levels). https://www.epa.gov/risk/regional-screening-levels-rsls-generic-tables-may-2016.

[9] Ministry of Housing Spatial Planning and Environment (1994). Environmental Quality Objectives in the Netherlands: A Review of Environmental Quality Objectives and Their Policy Framework in the Netherlands.

[10] Ministry of Housing and Urban-Rural Development (2009). Disposal of Sludge from Municipal Wastewater Treatment Plant-Control Standards for Agricultural Use (cj/t 309–2009).

[11] Schwarzenbach R.P., Gschwend P.M., Imboden D.M. (2003). Environmental Organic Chemistry.

[12] Wang X.-H., Wang X., Huppes G., Heijungs R., Ren N.-Q. (2015). Environmental implications of increasingly stringent sewage discharge standards in municipal wastewater treatment plants: Case study of a cool area of China. J. Clean. Prod..

[13] Zeng S., Chen X., Dong X., Liu Y. (2017). Efficiency assessment of urban wastewater treatment plants in China: Considering greenhouse gas emissions. Resour. Conserv. Recycl..

[14] Zhang M., Wang C., Peng Y., Wang S., Jia F., Zeng W. (2016). Organic substrate transformation and sludge characteristics in the integrated anaerobic anoxic oxic–biological contact oxidation (a2/o-bco) system treating wastewater with low carbon/nitrogen ratio. Chem. Eng. J..

[15] Ma Y., Peng Y., Wang X. (2009). Improving nutrient removal of the aao process by an influent bypass flow by denitrifying phosphorus removal. Desalination.

[16] Wu Q., Leung J.Y., Tam N.F., Chen S., Mai B., Zhou X., Xia L., Geng X. (2014). Biological risk and pollution history of polycyclic aromatic hydrocarbons (PAHs) in nansha mangrove, south China. Mar. Pollut. Bull..

[17] US Environmental Protection Agency Epa Method 8270d (sw-846): Semivolatile Organic Compounds by Gas Chromatography/Mass Spectrometry (gc-ms). https://www.epa.gov/sites/production/files/2015–07/documents/epa-8270d.pdf.

[18] Tsai P.-J., Shih T.-S., Chen H.-L., Lee W.-J., Lai C.-H., Liou S.-H. (2004). Assessing and predicting the exposures of polycyclic aromatic hydrocarbons (PAHs) and their carcinogenic potencies from vehicle engine exhausts to highway toll station workers. Atmos. Environ..

[19] Bortey-Sam N., Ikenaka Y., Nakayama S.M., Akoto O., Yohannes Y.B., Baidoo E., Mizukawa H., Ishizuka M. (2014). Occurrence, distribution, sources and toxic potential of polycyclic aromatic hydrocarbons (PAHs) in surface soils from the kumasi metropolis, ghana. Sci. Total Environ..

[20] Nguyen T.C., Loganathan P., Nguyen T.V., Vigneswaran S., Kandasamy J., Slee D., Stevenson G., Naidu R. (2014). Polycyclic aromatic hydrocarbons in road-deposited sediments, water sediments, and soils in sydney, australia: Comparisons of concentration distribution, sources and potential toxicity. Ecotoxicol. Environ. Saf..

[21] Zheng B., Wang L., Lei K., Nan B. (2016). Distribution and ecological risk assessment of polycyclic aromatic hydrocarbons in water, suspended particulate matter and sediment from daliao river estuary and the adjacent area, China. Chemosphere.

[22] Zhang L., Dong L., Ren L., Shi S., Zhou L., Zhang T., Huang Y. (2012). Concentration and source identification of polycyclic aromatic hydrocarbons and phthalic acid esters in the surface water of the yangtze river delta, China. J. Environ. Sci..

[23] US Environmental Protection Agency (1986). Guidelines for the Health Risk Assessment of Chemical Mixtures.

[24] US Environmental Protection Agency (1989). Supplemental Risk Assessment Guidance for the Superfund Program. Part 1. Guidance for Public Health Risk Assessments.

[25] Krishnan K., Paterson J., Williams D.T. (1997). Health risk assessment of drinking water contaminants in canada: The applicability of mixture risk assessment methods. Regul. Toxicol. Pharmacol..

[26] US Environmental Protection Agency (2000). Available Information on Assessment Exposure from Pesticides in Food.

[27] US Environmental Protection Agency (1986). Superfund Public Health Evaluation Manual.

[28] Qiao M., Qi W., Liu H., Qu J. (2014). Occurrence, behavior and removal of typical substituted and parent polycyclic aromatic hydrocarbons in a biological wastewater treatment plant. Water Res..

[29] Foan L., Domercq M., Bermejo R., Santamaria J.M., Simon V. (2015). Mosses as an integrating tool for monitoring PAH atmospheric deposition: Comparison with total deposition and evaluation of bioconcentration factors. A year-long case-study. Chemosphere.

[30] Azpíroz G., Blanco C.G., Banciella C. (2008). The use of solvents for purifying industrial naphthalene from coal tar distilled oils. Fuel Process. Technol..

[31] Tian W., Bai J., Liu K., Sun H., Zhao Y. (2012). Occurrence and removal of polycyclic aromatic hydrocarbons in the wastewater treatment process. Ecotoxicol. Environ. Saf..

[32] Ozaki N., Takamura Y., Kojima K., Kindaichi T. (2015). Loading and removal of PAHs in a wastewater treatment plant in a separated sewer system. Water Res..

[33] Bergqvist P.A., Augulytė L., Jurjonienė V. (2006). Pah and pcb removal efficiencies in umeå (Sweden) and šiauliai (Lithuania) municipal wastewater treatment plants. Water Air Soil Pollut..

[34] Vogelsang C., Grung M., Jantsch T.G., Tollefsen K.E., Liltved H. (2006). Occurrence and removal of selected organic micropollutants at mechanical, chemical and advanced wastewater treatment plants in norway. Water Res..

[35] Byrns G. (2001). The fate of xenobiotic organic compounds in wastewater treatment plants. Water Res..

[36] Manoli E., Samara C. (2008). The removal of polycyclic aromatic hydrocarbons in the wastewater treatment process: Experimental calculations and model predictions. Environ. Pollut..

[37] Man Y.B., Chow K.L., Cheng Z., Mo W.Y., Chan Y.H., Lam J.C., Lau F.T., Fung W.C., Wong M.H. (2017). Profiles and removal efficiency of polycyclic aromatic hydrocarbons by two different types of sewage treatment plants in Hong Kong. J. Environ. Sci..

[38] Ding J., Chen B., Zhu L. (2012). Biosorption and biodegradation of polycyclic aromatic hydrocarbons by phanerochaete chrysosporium in aqueous solution. Chin. Sci. Bull..

[39] Cai Q.Y., Mo C.H., Wu Q.T., Zeng Q.Y., Katsoyiannis A. (2007). Occurrence of organic contaminants in sewage sludges from eleven wastewater treatment plants, China. Chemosphere.

[40] Yunker M.B., Macdonald R.W., Vingarzan R., Mitchell R.H., Goyette D., Sylvestre S. (2002). Pahs in the fraser river basin: A critical appraisal of PAH ratios as indicators of PAH source and composition. Organ. Geochem..

[41] Budzinski H., Jones I., Bellocq J., Piérard C., Garrigues P. (1997). Evaluation of sediment contamination by polycyclic aromatic hydrocarbons in the gironde estuary. Mar. Chem..

[42] Zhang W., Wei C., Feng C., Yan B., Li N., Peng P., Fu J. (2012). Coking wastewater treatment plant as a source of polycyclic aromatic hydrocarbons (PAHs) to the atmosphere and health-risk assessment for workers. Sci. Total Environ..

[43] Katsoyiannis A., Terzi E., Cai Q.Y. (2007). On the use of PAH molecular diagnostic ratios in sewage sludge for the understanding of the PAH sources. Is this use appropriate?. Chemosphere.

[44] Tobiszewski M., Namiesnik J. (2012). Pah diagnostic ratios for the identification of pollution emission sources. Environ. Pollut..

[45] Mansuy-Huault L., Regier A., Faure P. (2009). Analyzing hydrocarbons in sewer to help in PAH source apportionment in sewage sludges. Chemosphere.

[46] Ministry of Health of the People’s Republic of China (1999). Environmental pollution health impact assessment standard. J. Environ. Health.

[47] Lee I.S., Sim W.J., Kim C.W., Chang Y.S., Oh J.E. (2011). Characteristic occurrence patterns of micropollutants and their removal efficiencies in industrial wastewater treatment plants. J. Environ. Monit..

[48] Qiao M., Qi W., Liu H., Qu J. (2014). Oxygenated, nitrated, methyl and parent polycyclic aromatic hydrocarbons in rivers of haihe river system, China: Occurrence, possible formation, and source and fate in a water-shortage area. Sci. Total Environ..

[49] Qi W., Liu H., Pernet-Coudrier B., Qu J. (2013). Polycyclic aromatic hydrocarbons in wastewater, WWTPs effluents and in the recipient waters of Beijing, China. Environ. Sci. Pollut. Res. Int..

[50] Zheng B., Ma Y., Qin Y., Zhang L., Zhao Y., Cao W., Yang C., Han C. (2016). Distribution, sources, and risk assessment of polycyclic aromatic hydrocarbons (PAHs) in surface water in industrial affected areas of the three gorges reservoir, China. Environ. Sci. Pollut. Res. Int..

